# Targeting the Bacterial Protective Armour; Challenges and Novel Strategies in the Treatment of Microbial Biofilm

**DOI:** 10.3390/ma11091705

**Published:** 2018-09-13

**Authors:** Nor Fadhilah Kamaruzzaman, Li Peng Tan, Khairun Anisa Mat Yazid, Shamsaldeen Ibrahim Saeed, Ruhil Hayati Hamdan, Siew Shean Choong, Weng Kin Wong, Alexandru Chivu, Amanda Jane Gibson

**Affiliations:** 1 Faculty of Veterinary Medicine, Universiti Malaysia Kelantan, Pengkalan Chepa 16100, Kelantan, Malaysia; li.peng@umk.edu.my (L.P.T.); anisa932@gmail.com (K.A.M.Y.); Shams88ns@gmail.com (S.I.S.); ruhil@umk.edu.my (R.H.H.); shean.cs@umk.edu.my (S.S.C.); 2 School of Health Sciences, Universiti Sains Malaysia, Kubang Kerian 16150, Kelantan, Malaysia; wengkinwong@usm.my; 3 UCL Centre for Nanotechnology and Regenerative Medicine, Division of Surgery & Interventional Science, University College London, London NW3 2PF, UK; a.chivu.14@ucl.ac.uk; 4 Royal Veterinary College, Pathobiology and Population Sciences, Hawkshead Lane, North Mymms, Hatfield AL9 7TA, UK; ajgibson@rvc.ac.uk

**Keywords:** biofilms, anti-biofilms, nosocomial pathogens, *Staphylococcus aureus*, *Pseudomonas aeruginosa*, *Klebsiella* pneumoniae

## Abstract

Infectious disease caused by pathogenic bacteria continues to be the primary challenge to humanity. Antimicrobial resistance and microbial biofilm formation in part, lead to treatment failures. The formation of biofilms by nosocomial pathogens such as *Staphylococcus aureus* (*S. aureus*), *Pseudomonas aeruginosa* (*P. aeruginosa*), and *Klebsiella pneumoniae* (*K. pneumoniae*) on medical devices and on the surfaces of infected sites bring additional hurdles to existing therapies. In this review, we discuss the challenges encountered by conventional treatment strategies in the clinic. We also provide updates on current on-going research related to the development of novel anti-biofilm technologies. We intend for this review to provide understanding to readers on the current problem in health-care settings and propose new ideas for new intervention strategies to reduce the burden related to microbial infections.

## 1. Microbial Biofilms and the Challenges in Infectious Disease

Bacterial infections remain a threat to human health despite the progress made in improving the quality of health care, and continuous development of antibiotics and vaccines to control disease. Bacterial infections can occur at any stage in human life and can often be controlled by a healthy immune system of the host. During hospitalization, patients are exposed to pathogen sources within the environment including medical equipment, other infected patients, and healthcare staff and are thus susceptible to hospital-acquired infections (HAI) [1]. Immunocompromised patients, the elderly or patients with existing chronic disease such as diabetes, cancer, cardiovascular diseases, or breakage of skin barrier such occurs with wounds, are reported to face a higher risk of nosocomial infection [2]. The typical medical device associated with HAI are related to central line bloodstream infections, ventilator pneumonia, and catheter urinary infections [1].

**Figure 1:**
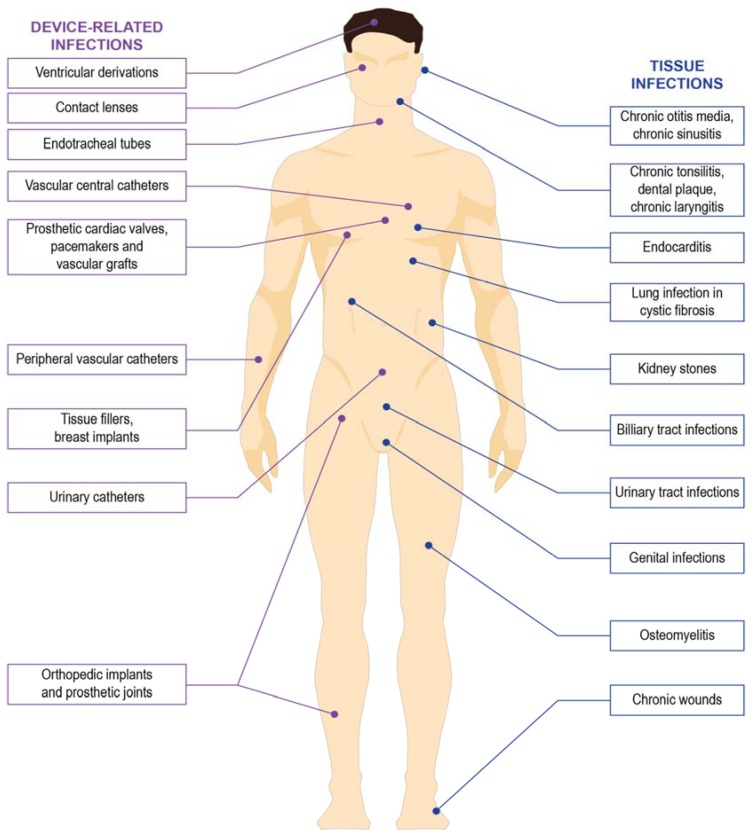
Common site of infections related to the formation of bacterial biofilms.

The increasing trend of nosocomial bacterial infections in immunocompromised hospitalized patients is worrying as HAI are the primary cause of morbidity in the health-care setting [3,4]. In the United States alone, it was estimated that a total of 1.7 million HAI occurred in the year 2002 alone, with 4.5 cases occurring for every 100 admissions, resulting in almost 99,000 deaths [5]. At the European level, more than 2 million patients contracted HAI, with 175,000 deaths per year [6]. The incidence of HAI are substantially lower at 7.1% and 4.5% in Europe and USA, respectively, compared to the low and middle-income countries (LMICs), where the average prevalence of HAI is 15.5% [7]. Among the leading bacteria that cause the HAI is *Staphylococcus aureus* (*S. aureus*), *Pseudomonas aeruginosa* (*P. aeruginosa*), *Escherichia coli* (*E. coli*), *Klebsiella pneumoniae* (*K. pneumoniae*), *Acinetobacter baumanii*, *Clostridium difficile*, and *Enterococci* [8,9]. The ability of these bacteria to form biofilms at the infected site or on medical devices has been increasingly recognized as one of the factors causing failure in the treatment of HAI, with biofilms estimated to contribute to approximately half of HAI [8,10]. Figure 1 shows the common site of infections related to the formation of bacterial biofilms.

### 1.1. *Methicillin-Resistant* Staphylococcus aureus (MRSA)

*Staphylococcus aureus* is a Gram-positive cocci-shaped bacterium that forms part of the normal flora on the body and is frequently isolated from the skin, respiratory tract, and female lower reproductive tract [11,12]. Infections by *S. aureus* were once treatable by penicillin. However, increasing resistance towards penicillin led to the introduction of methicillin for the treatment in 1960 [13]. However, soon after that, *S. aureus* acquired resistance towards methicillin, giving rise to methicillin-resistant *S. aureus* (MRSA) clones [12,13]. Currently, the Center for Disease Control (CDC) reported that infections by MRSA are the second most common cause of HAI in the USA [14]. The most common route of MRSA transmission is through direct contact. The ability of the organism to form biofilms on tissues such as the skin and inert indwelling device surfaces such as intravenous catheters and surgical implants further expose susceptible individuals [15]. Development of worldwide antibiotic resistance towards first-line therapies such as vancomycin and teicoplanin continues to hinder and restrict the successful treatment of MRSA infection [15]. Additionally, *S. aureus* also can invade host cells and evade the antimicrobial effects of administered therapies [16]. Together, these characteristics allow this organism to remain an important pathogen.

### 1.2. Pseudomonas aeruginosa

*P. aeruginosa* is a Gram-negative, rod-shaped, facultative anaerobe ubiquitous in the environment and forms part of the normal gut flora. Increasing resistance towards the multiple antibiotics, e.g., cephalosporins and carbapenem further compounds the problem due to the emergence of extremely drug-resistant (XDR) *P. aeruginosa* infections [17,18]. The last available treatment resort is colistin, a polymyxin antibiotic which was avoided for the past three decades as it may cause both neuro- and nephrotoxicity [19,20,21]. Patients dependent on breathing machines or fitted with an invasive device such as a catheter, are at risk of severe and life-threatening illness from *P. aeruginosa* capable of forming biofilms on medical device surfaces [22,23,24,25]. *P. aeruginosa* biofilms were reported to cause endocardial valve infection through endocardial tubes, ventilator-associated pneumonia (VAP), and catheter-associated urinary tract infections (CAUTI). Additionally, *P. aeruginosa* has also been reported to be able to grow in intravenous fluid and could enter the bloodstream and cause sepsis [26,27,28].

### 1.3. Klebsiella pneumoniae

*Klebsiella pneumoniae* (*K. pneumoniae*) is a non-fastidious, Gram-negative bacillus, and is usually encapsulated. The bacterium is one of the normal flora present in the mouth, skin, and intestine, yet it has been reported to cause pneumonia, urinary tract infections, and bacteremia in patients from hospitals, nursing homes, and other healthcare facilities [18]. *K. pneumoniae* is known as a remarkably resilient pathogen as it can evade and survive rather than actively suppress many components of the immune system of the infected host [29]. The bacteria have developed resistance towards almost all available antibiotics; fluoroquinolones, third-generation cephalosporins and aminoglycosides [30]. Recently, the emergence of the carbapenem-resistant *K. pnemoniae* strains which currently circulating across the globe has forced the administration of colistin, an old and considered the last available antibiotic [31]. Additionally, resistance towards colistin was recently reported, showed that the bacteria are capable of evading all types of available antibiotics, leaving no drugs left for the treatment [32]. Compounding the problem, this organism can survive and grow within the intravenous fluid and form biofilm on medical devices such as the urinary catheter, leading to detrimental septicemia in patients [26,33,34,35].

## 2. The Physiology of Biofilms

### 2.1. Definition and the Structure of the Biofilm

**Figure 2:**
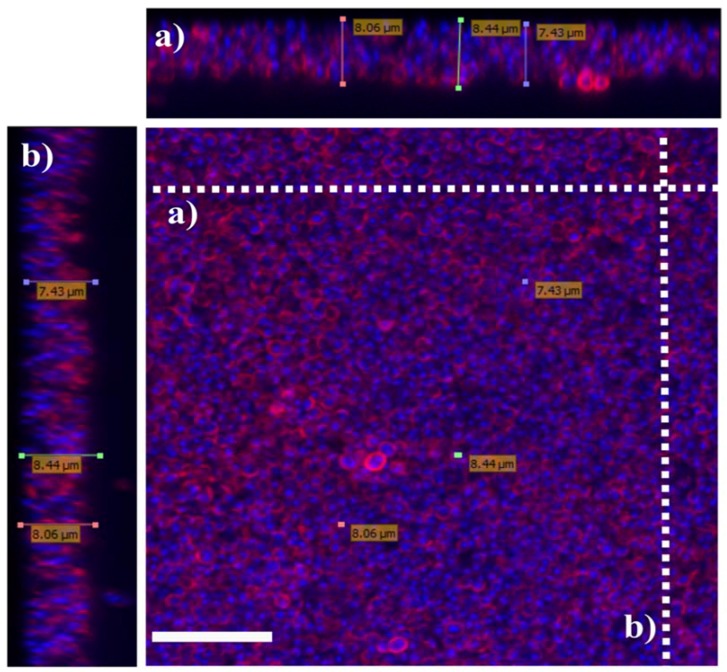
The three-dimensional structure of *S. aureus* biofilms visualized with a confocal microscope.

Biofilms have been described in environmental and technical microbiology for more than 90 years. However, the importance of microbial biofilms in medicine has only been recognized since the early 1980s [36]. A microbial biofilm is defined as a structured consortium of microbial cells surrounded by the self-produced matrix [37]. The rough structure can be described as polymicrobial aggregates that resemble mats, flocs, or sludge that accumulate at interfaces. Because of the soft and fragile structure, it is difficult to physically characterize the structure from the infected or adhered surfaces in-vivo. Thus, characterizations of biofilm were mostly performed using in-vitro cultured biofilm cells. The thickness of biofilms can vary depending on the species of the bacteria, the duration and the method used to grow the biofilm. In-vitro, the biofilms of *P. aeruginosa* can reach 209 µm; *S. aureus*, 8.0 µm; and *K. pneumoniae*, 231 µm [33,38,39]. Figure 2 shows the three-dimensional structure of *S. aureus* biofilms visualized with a confocal microscope with the thickness of approximately 8.0 µm.

Biofilms are built of planktonic (individual) bacterial cells ‘glued’ together with self-released extra-polymeric substances (EPS) which consists of lipopolysaccharides, proteins, lipids, glycolipids, and nucleic acids [40]. The type of polysaccharides found within the biofilm depends on the bacteria species, *S. aureus* and *S. epidermidis* produce poly-ß(1,6)-*N*-acetyl-d-glucosamine (PNAG) [41], while *P. aeruginosa* produces Pel (a cationic exopolysaccharide composed of 1–4 linked galactosamine and glucosamine sugars) and Psl (a penta-saccharide composed of d-glucose, d-mannose and l-rhamnose) [42,43]. The nucleic acid found in EPS is known as extracellular DNA (eDNA), generated by lysis of a subpopulation of the same bacteria under the control of quorum sensing, a mode of communication between cells. The role of EPS is to promote adhesion and aggregation of bacteria to the surfaces and provides stability to the biofilm structure. Direct interaction of eDNA with extracellular calcium (Ca^2+^) within the biofilm induces bacterial aggregation via cationic bridging. The positive charge of the extracellular Ca^2+^ modifies the bacterial cell surface charge by neutralizing the electrostatic repulsion between negatively charged biofilm components, thus assisting cellular aggregation and adherence of bacteria to material and tissue surface [44,45]. Once the biofilm is established, negatively charged eDNA acts as a biofilm defence mechanism by chelating the cationic antimicrobial peptides from the host immune system. eDNA also chelates divalent cations triggering the bacterial transcription of genes responsible for increasing pathogenicity and resistance to antimicrobials [46].

*S. aureus* biofilms were cultured in tryptic soy broth for 48 hours, fixed with 4% paraformaldehyde and treated with wheat germ agglutinin (WGA, red) to stain n-acetylglucoseamine component of polysaccharide and DAPI (blue) to stain the bacteria nuclear material, followed by confocal microscopy z-stack projection that moved through 111 slices across the cell. (a) Horizontal cross-section of biofilms and (b) Vertical cross-section of biofilms. White scale bar is 7.5 µm. The approximate thickness of the biofilms was 7.9 ± 0.5 µm. Image adapted from Kamaruzzaman et al., 2017 [39].

### 2.2. Development of Biofilms

**Figure 3:**
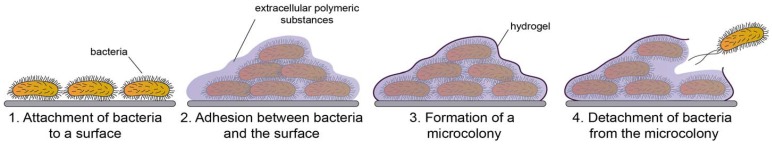
Stages in biofilm information The image is adapted from [50].

Development of biofilms generally involves several stages, represented schematically in Figure 3. (1) Attachment of cells to surfaces: In this step, bacteria use eDNA and organelles and proteins (such as flagella, pili, fimbriae, and outer membrane proteins) that can assist in sensing and attaching to surfaces [47]; (2) Adhesion between cells and surfaces: Here, the EPS components consisting of DNA, lipoproteins, and lipids secreted by the bacteria encourages cell to cell and cell to surface adhesion; (3) Replication of cell and formation of microcolonies: In this stage, the bacteria become encapsulated in a layer of hydrogel, which functions as a physical barrier between the community and the extracellular environment. The bacteria within the community communicate with each other through quorum sensing (QS), a chemical communication chemical signal that modulates cellular functions, including pathogenesis, nutrient acquisition, conjugation, motility, and production of secondary metabolites [48,49]. Within this stage, the biofilm will mature as cells replicate and EPS accumulates; (4) Cell detachment from biofilms: The final stage involves the detachment of bacterial cells from the microcolonies. Cells are then capable of forming a new biofilm colony at another location [50,51].

### 2.3. Mechanism of Antibiotic Resistance in Microbial Biofilms

Bacterial cells existing as biofilms can be 10–1000 times more resistant to antibiotics [52,53,54]. This condition is attributed by several factors and briefly summarised in Table 1. An excellent review on detailed mechanism of biofilm mediated antimicrobial resistance and tolerance was recently published by Hall & Mah (2017), thus readers are invited to refer to this article for detailed information [55].

### 2.4. Immune Evasion of Biofilms

Bacteria within the biofilm can evade recognition by the innate immune system and thus avoid being eliminated or controlled by the immune system. While the purpose of this article is not to provide a comprehensive review of immune evasion of biofilms, there are aspects worth comment in the context of novel therapies and control strategies. We direct the reader to reviews by Roilides et al., Le et al., and Gunn et al. for more extensive information [64,65,66]. Despite a clear shortage of studies concerning biofilm recognition by the immune system compared to planktonic cells, several common evasion mechanisms have been described. Biofilms evade the natural antimicrobial properties of innate immune cells by thwarting pathogen recognition, phagocytosis, and cellular activation [67]. In some cases, the cells of the innate immune system assist the process of biofilm growth and maturation [68].

Biofilms modulate leukocyte function by reducing phagocytosis, despite active migration of cells towards the biofilm. Phagocytosis rates of biofilm material are thought to be diminished through a lack of microbial recognition by pattern recognition receptors due to concealed or obscured bacterial ligands such as lipoproteins, lipopolysaccharides, and nucleic acids (i.e., though EPS protection) [69]. EPS protection also retains bacterial content within the biofilm, resulting in large complexes which leukocytes are incapable of engulfing. Detached or homogenised (via sonication) bacteria from biofilms are readily phagocytosed suggesting that leukocyte modulation is temporary [67]. Leukocyte antimicrobial function and killing via oxidative burst (production of reactive oxygen species and nitric oxide) is generally considered to be greatly reduced when considering responses against biofilms [70]. Oxidative burst is readily linked to reduced receptor mediated recognition, further highlighting the role of the protective function of the biofilm matrix in immune evasion. 

Experimental investigations with relevant HAI associated biofilms such as those of *S. aureus* and *P. aeruginosa* continue to enhance the knowledge surrounding immune recognition in the context of biofilms. Neutrophils are attracted to and migrate towards both *S. aureus* and *P. aeruginosa* biofilms upon which a reduced phagocytic behaviour is observed along with decreased microbial killing [67]. Lack of *S. aureus* recognition by pattern recognition receptors known as Toll-like Receptors (TLR) is likely due to ligand inaccessibility within the biofilm [69]. TLR binding and subsequent activation potentiates phagocytosis and antimicrobial activity through oxidative burst mechanisms, thus the arrangement of biofilms within a protective matrix serves to conceal internal components. Disruption of *P. aeruginosa* biofilms to homogenised individual cells exhibited an increase in phagocytosis demonstrating the importance of the protective nature of the biofilm matrix [67].

Understanding fundamental interactions between biofilms and the immune system presents opportunities for novel control strategies. Glycolipids involved in quorum sensing produced by *P. aeruginosa*, rhamnolipids, protects against neutrophil activity by inducing lytic necrosis [71]. Disruption of this protection via mutation or quorum inhibitors increases phagocytosis rates, thus targeting active evasion mechanisms offers a route for innovative biofilm treatment strategies.

## 3. Guideline for Management of Biofilm Associated Infection

Tackling the central issue of HAI biofilms requires that diagnosis and treatment be collectively examined (in a concerted manner) and selected to avoid senseless efforts and increase chances for effective therapy. Several guidelines and recommendations are available for diagnosis and treatment of biofilm infection [72,73] and are summarised in Table 2.

## 4. Diagnosis of Biofilm Mediated Infections

The diagnosis of biofilm-related infections is complex and should combine different approaches and a multidisciplinary perspective from clinical and laboratory diagnosis [74]. The European Society for Clinical Microbiology and Infectious Disease (ESCMID) has provided a detailed guideline to aid clinicians in the diagnosis of biofilm related infections. Briefly the guideline emphasizes the combination of clinical and laboratory diagnosis to facilitate clinicians in providing an effective therapy to patients [73]. Generally, the clinical diagnosis may include clinical signs, case history, failure of antibiotic therapy and persistent infection. For laboratory diagnosis, the following techniques have demonstrated high efficacy, successfully discriminating between planktonic and biofilm mediated infections.

### 4.1. Sonication 

Sonication is a process that uses acoustic energy to agitate particles in a solution. This method is useful and recommended to be applied to remove microbial biofilms from the biomaterial surface [75]. The suspected source of infection (e.g., implant) is removed from the patient and subjected for sonication. The process disrupts strongly adhered biomass and releases the biofilm into the solution allowing further analysis to further identify the microbial species [76]. Sonication has been shown to be useful in removing biofilms from urinary catheters, cardiac implantable electronic devices, and prosthetic implants [75,76,77].

### 4.2. Polymerase Chain Reaction (PCR)

PCR is one of the common methods used for detection of pathogens directly from clinical specimens. The method is based on the amplification of specific conserved regions within the targeted organism, providing high specificity and sensitivity in discriminating species of bacteria. Due to the robustness of the method, PCR-based diagnostic tests have been used for detecting a range of pathogens including bacteria, viruses, parasites, and fungi [78,79,80,81]. In biofilm diagnosis, PCR has been applied to detect the microbial species that forms biofilms on tissues or biomaterials following the sonication process. For example, PCR was used to detect coagulase negative staphylococci, *S. aureus*, *Cutibacterium* species, *Enterococci*, and *Candida* spp. following sonication of implants in the case of orthopedic hardware associated infections [77].

### 4.3. Matrix-Assisted Laser Desorption Ionization Time-of-Flight Mass Spectrometry (MALDI-TOF MS)

In recent years, matrix-assisted laser desorption ionization time-of-flight mass spectrometry (MALDI-TOF MS) devices have become widely available, changing laboratory workflows for identification of pathogens in most clinical microbiology laboratories [82]. The bacterial protein profiles obtained from intact or cell extracts can be compared to a database of bacterial reference mass spectra for rapid identification at the genus, species, and subspecies level [83]. Also, the method allows for differentiation between planktonic and biofilm cells, as these two populations display different spectra. The method has been tested in different species of Gram-positive and negative bacteria [84].

### 4.4. Fluorescence In Situ Hybridization (FISH)

FISH is a method that involves binding of short (between 18–25 base pairs), fluorescently labeled oligonucleotides that can bind to the specific ribosomal RNA of the target organism (bacteria, protozoa, and yeast). Analysis involves visualization of targeted ribosomal RNA by fluorescence microscopy [85]. This method can be applied directly on intact specimen fragments, without the need for time consuming sub-culture. Identification of biofilms using FISH is further enhanced by the ability to easily detect bacterial aggregation. The method has been successfully applied to detect biofilm of the following bacteria: *Streptoccous* spp.; *S. aureus*; *Gardnerella vaginalis*; and *Atopobium vaginae* in vaginal biofilms [85,86,87,88].

### 4.5. Microscopy

Microscopy is commonly used following the sonication or FISH to visualize the bacterial aggregates in the specimen. Bacterial growth within biofilms are commonly found as aggregates compared to individual dispersed cells observed during the growth of planktonic cells [85]. The most common microscopy technique used to visualize microbial biofilms are scanning electron microscopy (SEM), transmission electron microscopy (TEM), and scanning confocal laser microscopy (SCLM). The high resolution provided by these techniques allows for depth analysis within the biofilm structure and thus assists the diagnosis of infection mediated by microbial biofilm [89,90,91]. Table 3 summarizes the common laboratory techniques employed to assist the diagnosis of biofilm mediated infections.

## 5. The Potency of Existing Therapies against Microbial Biofilm

Existing antibiotics are useful resources for the treatment of infections with potential formation of biofilm and are summarized in Table 4. Application of suitable antibiotics that are effective against biofilm can improve the outcome, reduce relapse, and improve recovery of the patients. Effective eradication of biofilms could be achieved with high doses of antibiotics, combined with drugs that weaken the biofilm. Efficient treatment of biofilm infections demands a well-established multidisciplinary collaboration which includes: removal of the infected foreign bodies, selection of biofilm-active and -penetrating antibiotics, systemic or topical antibiotic administration in high dosage combinations, and administration of anti-quorum sensing or biofilm dispersal agents [72]. Specifically, treatment of biofilms requires effective and well-penetrating antibiotics to ensure a sufficient concentration of effective antibiotic at the site of biofilm infection. In general, macrolides, lincosamides, tetracyclines, rifamycins, quinolones, fusidic acid, nitroimidazole, sulfonamides, and oxazolidinones penetrate better in tissues and cells than beta-lactam (including penicillins, cephalosporins, and carbapenems), aminoglycosides, glycopeptide, and polymyxin [72]. Antimicrobial agents such as meropenem, colistin, and azithromycin are good candidates for combination strategies which maintain their activity under conditions of reduced oxygen tension and low metabolic activity as found in deeper layers of biofilms. Some of the widely used antibiotics such as azithromycin, ceftazidime, and ciprofloxacin have Quorum Sensing Inhibitor (QSI) activity in addition to their conventional antibiotic activity. An example of a drug that can be included in combination approaches for *P. aeruginosa* biofilm infection is azithromycin. The drug has been shown to inhibit or reduce the production of several of the virulence factors (elastase and rhamnolipids) of *P. aeruginosa*, as well as the matrix component alginate as well as inhibiting quorum sensing. More drugs like azithromycin are required, ideally with higher potency [95].

The current combination regimen recommended for the treatment of carbapenem-resistant *K. pneumoniae* (CR-KP) includes high-dose carbapenem therapy (first-line antibiotic), which is combined with colistin and or tigecycline, gentamicin, or fosfomycin (second-line antibiotics). Synergistic interactions between the first-line and second-line antibiotics minimize the use of extremely high doses and the emergence of resistance, as well as potentiate the effectiveness of individual agents. Rifampicin is also occasionally considered for inclusion in combination regimens because of its ability to penetrate intracellular sites and biofilms, which could be important in the treatment of CR-KP infections involving prosthetic material. A new antimicrobial with activity against CR-KP, ceftolozone, is a potent new cephalosporin that is not degraded by current AmpC cephalosporinases or affected by known porin mutations and efflux pumps circulating in CR-KP strains. When tested in combination with tazobactam, the drug has demonstrated promising activity in-vitro against multi-drug resistant Gram-negative isolates, including CR-KP [72].

## 6. Promising Novel Therapies for Prevention and Treatment of Biofilm Associated Infections

### 6.1. Nanoparticles

Nanoparticles are particles that have an internal structural measurement or external dimensions within the nanometers size and can be acquired from metallic, metal oxide, semiconductor, polymer, or carbon-based materials [16,106]. There are two major groups of nanoparticles, organic (e.g., micelles, dendrimers, liposomes, hybrid, and compact polymer) and inorganic nanoparticles (e.g., fullerenes, quantum dots, silica, gold, and graphene) [107]. Nanoparticle-mediated antibacterial activities depend on the composition, surface modification, intrinsic properties, and the bacterial species [108]. The reported mechanisms of antibacterial activities include disruption of the bacterial membrane, condensation of the bacterial genome and induction of reactive oxygen species that can be harmful to the physiology of the bacteria [109,110,111]. Graphene, one of the recently discovered nanoparticles, exhibits antibacterial activity through direct interaction of the compound with the bacterial membrane, causing stress to the membrane, releasing the intracellular contents and bacterial cell death [107]. 

Additionally, nanoparticles are reported to have anti-biofilm activities by effectively removing and preventing formation of biofilm on surfaces. Table 5 summaries nanoparticles with anti-biofilm activities. The mechanisms of nanoparticle anti-biofilm activity have only been partially understood. For example, silver nanoparticles mediate anti-biofilm activity by suppressing the polysaccharide intercellular adhesion (PIA) synthesis, thus preventing bacterial adhesion to surfaces [112]. On the other hand, zinc oxide (ZnO) prevents biofilm formation by inhibiting microbial growth and prevents biofilm to reach a steady state [113]. Additionally, Yadav et al. demonstrated that graphene oxide coated on surfaces reduced attachment of bacteria, thus preventing biofilm formation [107].

Application of nanoparticles as an antibacterial provides several advantages compared to conventional antibiotics. Nanoparticles have multiple modes of action and range of bacterial targets which would require several bacterial mutations to resist antimicrobial activity. As such nanoparticles present a considerable advantage over conventional antibiotics as they offer an option where there is a reduced likelihood to develop resistance based on intrinsic properties [114]. This is especially important when there is an increase of bacteria resistance of new strains against most potent antibiotics.

### 6.2. Diterpenoids

Diterpenoids are a group of plant-derived metabolites composed of two terpene units with molecular formula C_20_H_32_. Based on number of rings present in the molecular structure, diterpenoids were classified into acyclic (e.g., phytanes), monocyclic (e.g., Cembrene A), bicyclic (e.g., labdanes, halimanes, and clerodanes), tricyclic (e.g., pimaranes, abietanes, cassanes, and dehydroabietic acid), tetracyclic (e.g., trachylobanes and kauranes,), and macrocyclic diterpenes (e.g., taxanes, cembranes) [118]. These metabolites have shown potential values to substitute antibiotics for the treatment of antibiotic-resistant and biofilm forming microbes such as *S. aureus*. Abietane-type diterpenoids extracted from the root of *Salvia sclarea*, a medicinal plant used for easing stomach ache, diarrhoea, sore throat swelling, and headaches, revealed microbicidal and microbiostatic activity against *S. aureus* as well as acanthamoeba, a free-living amoeba. The active compound, salvipisone, showed potential anti-biofilm activity against the antibiotic-resistant Staphylococci, greater than most reported antibiotics [16]. Dehydroabietic acid (DA), an abietane-type diterpenoid found in the resin of coniferous trees, is effective in the inhibition of *S. aureus* biofilm formation in the low molar range, while the effective dose that reduced the viability and biomass of the formation of a biofilm was just two to four-fold higher than the inhibitory dose [119]. Hybrids of DA and selected amino acids resulted in potent fast-acting disassembly of biofilms and weakened the integrity of the bacterial membrane. Also, the DA-amino acid hybrids are potentially more resistant against proteolysis as compared to DA alone. The concentration of bactericidal dose is only three to six folds higher than the bactericidal dose [120]. 8-hydroxyserrulat-14-en-19-oic acid, a plant-derived serrulatane diterpenoid (extracted from the Australian medicinal plant *Eremophila neglecta*) showed anti-biofilm formation activity against Gram-positive bacterial but not Gram-negative bacteria by inhibiting the macromolecular biosynthesis of the bacterial membrane [121]. Table 6 summarises examples of diterpenoids with anti-biofilm activities

### 6.3. Biomacromolecules

Biomacromolecules such as polysaccharides, naturally secreted polymers, aliphatic, cyclic, and aromatic organic acids were studied for their potential in preventing and constraining biofilm formation or resolving the formed biofilm. Distinct from antibiotics, these macromolecules do not target specific intracellular molecules within the microbes. Instead, with their amphiphilic characteristics, the cationic group of the molecules facilitates microbial targeting and water solubility; while the hydrophobic group induces membrane lysis; being attracted to the microbial membrane via interaction between their cationic groups and the anionic membrane’s surface. Upon physical interaction, the hydrophobic group penetrates the microbial membrane leading to membrane destabilization and cytoplasmic content leakage. Antimicrobial agents with physical membrane disruption mechanisms are less likely to be targeted by antimicrobial resistance [122]. Polysaccharides produced by a bacterium may affect biofilm formation of other species through competition and cooperation phenomena [123]. For instance, *K. pneumoniae* capsular polysaccharide was suggested to restrict biofilm formation in several clinically important Gram-positive and Gram-negative bacterial species such as *S. epidermidis*, *S. aureus*, *E. coli*, and *Enterobacter aerogenes* [124,125]. Extracellular polymeric substances such as natural high molecular weight polymers secreted by bacteria have also been suggested to be anti-biofilm candidates [126]. Interruption of bacterial signaling systems such as quorum sensing affects bacterial biofilm formation. In case of a mixed culture, acyl-homoserine lactonase produced by *Bacillus cereus* showed inhibition and settlement of *Vibrio cholera* biofilm [127]. On the other hand, commercial organic acid products usually used in food industry showed the ability to reduce *Salmonella enterica* viable count and biofilm post-treatment, but not total elimination of the bacterium [128]. 

Incorporating macromolecules with anti-biofilm properties onto the surface of biomaterials has gained increasing interest, especially in implant-associated medical devices such as intravascular catheters, urinary catheters, and orthopedic implants [129]. Coating of biomaterial surface with natural or modified polysaccharide polymers such as hyaluronic acid, heparin, and chitosan revealed promising findings in battling implant-associated biofilm infections [130]. Table 7 summarises examples of examples of biomacromolecules with anti-biofilm activities.

### 6.4. Honey

Honey, an ancient wound remedy has gathered renewed interest in its clinical potential for inhibiting a wide range of infectious agents and promoting rapid wound healing [131]. The availability of medical grade honey with laboratory proven effects at the cellular and molecular level against certain microorganisms is not uncommon [131,132,133,134]. Mechanisms of bacterial inhibition within biofilm formation attributed to honey is a particular focus due to the increasing multidrug resistance of biofilm-associated organisms. Fortunately, there is accumulating evidence to show honey displays activity in both preventing the formation of a biofilm either through interfering with adherence to host cells or interfering with quorum sensing and disrupting an established biofilm. Several types of honey have been shown to exhibit anti-biofilm activity in-vitro (Table 8).

Besides being used alone, honey also exhibits synergistic activity in combination therapy with certain antibiotics for planktonic cells [135,136,137]. Although promising, the application of honey as an antibacterial, anti-biofilm, and wound healing promoting alternative is still primarily confined to in-vitro testing. This is mainly due to the varying reports describing different sources of honey, the strain of bacteria, biofilm stage, optimum dosage used, and wound/biotic conditions that might influence the effectiveness of honey when being applied. A few in-vivo model using merino sheep and albino mice demonstrated that anti-biofilm activity was found in honey used, but advised that optimal clinical application should be titrated carefully as tissue toxicity and rejection of the necrosed area from the epidermis was also found with increasing concentration [138,139]. However, care also should be taken on the biofilm-enhancing action of low doses (<MIC) of honey that could be due to a stress response, which has been observed when bacteria in biofilms are exposed to sub-inhibitory concentrations of antibiotics [140]. 

### 6.5. Antimicrobial Peptides

Antimicrobial peptides (AMPs) are peptides molecules which are produced by many tissues and cell types in a variety of invertebrate, plant, and animal species. The natural peptides are generally made up of 10–50 amino acid residues, positively charged (+2 to +9), with around 50% of hydrophobic properties and diverse sequences and structures [153]. The antimicrobial activities of these peptides are attributed by the amino acid composition, ampipathicy, cationic charge, and size that allow them to attach and insert themselves into the bacterial membrane bilayers to form pores and thus, kill the bacteria [154]. Other than working effectively against planktonic bacterial cells, AMP has also been shown to be effective against biofilms. Mataraci, 2012 evaluated the anti-biofilm activities of two AMPs, indolicin and CAMA: cecropin (1–7)-melittin A (2–9) amide which was found to inhibit MRSA biofilms formation [9]. Additionally other AMPs are currently highlighted as a promising approach to prevent biofilm formation or to treat established biofilms, for instance, LL-37, HBD3, hep-20, IDR-1018 are able to inhibit several species of biofilm formation by either down-regulating the genes essential for biofilm development or up-regulate the expression of genes resulting in a marked attenuation of biofilm production and even by altering the architecture and reducing the amount of extracellular matrix [155,156,157]. AMPs also are known as host defense peptides are essential components of innate immunity in higher organisms, contributing to the first line of defense against infections [158]. While investigating possible anti-biofilm peptides from natural resources, synthetic peptides produced either by de novo synthesis or by modification gained increased interest based on their improved biological functions and reduced size, which in turn reduces production costs [159,160]. A curated list of useful AMP along with their antimicrobial properties has been documented by de Luca and held within a database called BaAMP accessed via www.baamps.it [161]. 

**Figure 4:**
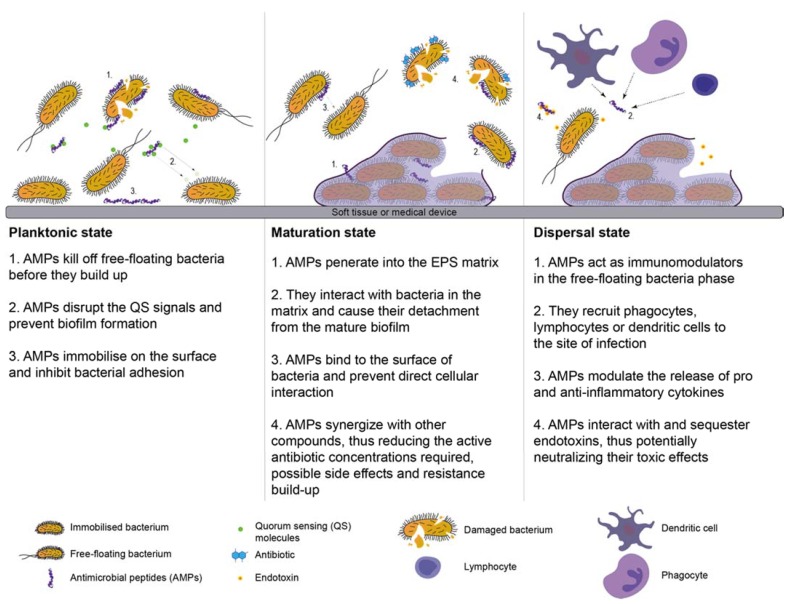
The three main steps of the biofilm life-cycle (attachment to a surface, maturation, and dispersal) and the mechanisms exhibited by Antimicrobial peptides (AMPs) in every step.

Several comprehensive works reviewing various aspects of AMPs have been performed [155,156,157]. Success of AMPs in both antimicrobial and anti-biofilm activities are collectively due to several characteristics namely: (i) rapid bactericidal effects; (ii) high plasticity in different microenvironments; (iii) good penetration into the matrix of extracellular polymeric substances (EPS); (iv) anti-quorum sensing; (v) host response modulator; and (vi) synergistic effects with other conventional and unconventional antimicrobial compounds [155]. The mechanisms exhibited by AMPs are illustrated in a different state in Figure 4.

### 6.6. Antimicrobial Polymer

Antimicrobial polymers are synthetic polymers covalently linked with functional groups with high antimicrobial activity such as amino, hydroxyl, and carboxyl groups [162]. Antimicrobial polymers are effective against a range of bacteria including the bacteria commonly associated with HAIs [163]. Due to the long and repeating chain of active and charged functional groups, the common mechanism of antimicrobial activity is through disruption of the cell wall or cytoplasmic membranes. Takahashi et al. demonstrated that cationic homopolymer PE0 and copolymer PE31 containing 31% of ethyl methacrylate was effective in removing biofilm of *Staphylococcus mutans* (*S. mutans*) compared to chlorohexidine and the cationic surfactant that was tested at the same concentration [162]. Another study by Li et al. showed that cationic monomer, methacryloxylethylcetyl dimethyl ammonium chloride (DMAE-CB) was also effective in the removal of a biofilm of *S. mutans*, the common bacteria associated dental problems [164]. Peng et al. modified the polyurethane compound, the main compound for catheter by copolymerization of an amine functionalized N-substituted diol to give a cationic polyurethane (Tecoflex-NH3), and showed that the cationic polyurethane shown contact killing of *E. coli* and prevent build-up of biofilm on the surfaces, thus, reducing the chances of CAUTI, one of the main causes of the nosocomial infections [165]. Another example of an antimicrobial polymer is Polyhexamethlene biguanide (PHMB), a cationic polymer that has been used in the clinic for over than 40 years with no sign of bacterial resistance [166]. PHMB mediates antibacterial activities through disruption of the cell wall and condensation of the bacterial chromosome [111], and recent discovery demonstrate PHMB efficacy in killing intracellular bacteria [16,167]. PHMB is also effective against biofilms from a range of bacterial species and thus effective when applied for the treatment of wound infections [39,168,169]. Though there are increasing findings for polymer mediated anti-biofilm activities, the complete mechanism of the activities is not fully understood. The anti-biofilm activities posed by the polymers could possibly be due to the interaction of the charged group on the polymer structure with the eDNA or Ca^2+^, thus disrupting the biofilm structure and cause destabilization. 

## 7. Summary and Outlook

Biofilms, a form of protective armour for the bacteria, are generally more resistant to the treatment. Biofilms will remain a challenge for the prevention and control of infection; thus it is critical that we continue to explore and understand the physiology and structure of biofilms to develop an improved, innovative, and novel targeted therapies. Based on promising in-vitro studies and reports investigating the use of nanoparticles, AMPs, diterpenoids, and biomacromolecules; we propose that these compounds should be the focus of future novel biofilm control strategies. Further studies focusing on the efficacy and tolerability in-vivo are required to ascertain the level of translation of in-vitro results to clinical resolution of infections caused by biofilms. Together with the increased understanding of the fundamental biology of biofilms, the application of novel or repurposed compounds will undoubtedly improve the prospect of treating and resolving biofilm infection within clinical settings.

## Figures and Tables

**Table 1:** Mechanism of biofilm-mediated antimicrobial resistance.

Physiology of Biofilm	Mechanism of Antimicrobial Resistance	References
Component of the biofilm matrix		
Thick biofilm matrix	Reduced permeation of antimicrobials across biofilm matrix	[52,54,56,57]
Expression of polysaccharide subunits in biofilm matrix	Expression of PsI polysaccharides in *P. aeruginosa* sequesters antimicrobials in the biofilm matrix via electrostatic interaction	[42]
Expression of antimicrobial modifying enzymes in the biofilm matrix	Beta lactamases produced by *Klebsiella pneumoniae* biofilm degrade beta lactam antibiotics	[58]
Expression of eDNA	eDNA interacts with antimicrobials and prevent further penetration of the agent across the biofilm matrix to reach cellular targets	[59]
Nutritional factors		
Oxygen deprived environment	Deeper layer of the biofilm is oxygen deprived (hypoxic). Hypoxic conditions reduce outer membrane potential of bacteria, diminishing intracellular transport of antibiotics into the bacterial cells Hypoxia induces increased (or is new expression?) expression of bacterial membrane efflux pumps, potentially reducing antimicrobial accumulation in the cell	[60,61,62]
Amino acid deprived environment	Amino acid starvation activates a stringent response in bacteria within biofilm increasing antimicrobial tolerance	[57]
Physiology of bacteria		
Small colony variants and persister cells	Surviving bacteria within the biofilm may change into small colony variants and persister cells. These variants present different phenotypes compared to the wild type planktonic cells including greater tolerance towards antimicrobials	[63]

Table adapted from Hall & Mah (2017) [55].

**Table 2:** Summary of guidelines for diagnosis and treatment of biofilm infections.

Steps	Detail Action
Biofilm diagnosis	Sampling on tissue biopsy or device/prosthesis (foreign bodies). Microbial cultivation and identification. Antibiotic susceptibilities test.
Reporting of biofilm associated infections	Biofilm associated infections may be reported using descriptive terms. 1. Detected e.g., ‘Microscopy shows Gram-negative rods in biofilm-like structures’ 2. Suspected e.g., ‘Growth of/PCR-detected microorganisms possibly from a biofilm infection’
Treatment of biofilm related infections 1. Removal sources of infections	Debridement of tissues Removal of foreign bodies
2. Administration of topical antiseptics	Empiric therapy to prevent biofilm formation after debridement of tissues Pre-emptive therapy to prevent possible biofilm reformation when specific bacteria is detected from the debride tissue
3. Administration of antibiotics	Selection of antibiotics suitable for biofilm associated infections Administration of antibiotics Optimization of dosage
Monitoring	Assessment of wounds Appropriate/maintenance debridement Re-evaluation of antiseptics/antibiotics efficacy
Standard care	Set-up the advance therapies Incorporation of novel techniques

Diagnosis and treatment of biofilm can be achieved by various methods, approaches in diagnosing and treating biofilm infections will be further discussed in the following sections.

**Table 3:** The common laboratory techniques applied for laboratory diagnosis of biofilm mediated infections.

Biofilm Mediated Infections	Bacterial Species	Techniques	References
Chronic otitis media	*S. pneumoniae*, *M. catarrhalis*, *P. aeruginosa*, *S. aureus*, and *K. pneumoniae*	PCR and SEM	[92]
Periprosthetic Infection	*Coagulase-negative Staphylococci*, *Propionibacterium* spp., *Streptococci* and *Enterococci*	Sonication & PCR	[93]
Chronic wound	*P. aeruginosa* and *S. aureus*	TEM & FISH	[93]
Catheter-associated infection	*E. coli*	Sonication & SEM	[76]
Chronic rhinosinusitis	*S. aureus* and *P. acnes*	FISH	[94]
Cystic fibrosis	*P. aeruginosa*	FISH and light microscope	[93]

**Table 4:** Summary of existing antibiotic regimens according to the specific microbial species.

Bacteria	Biofilm Site of Infection	Antibiotic Regimen	Duration	Route of Administration	References
*P. aeruginosa*	Lung infection in cystic fibrosis (CF)	0.5–2 MU colistin, twice daily	Continuous	Inhalation	[96]
		300 mg tobramycin, twice daily	28 days on/off cycles		
		75 mg aztreonam, three times daily	28 days on/off cycles		
		32.5 mg or 65 mg ciprofloxacin, once daily	28 days		
	Lung infection in non-CF bronchiestasis	1 MU colistin, twice daily	Continuous	Inhalation	[97]
		32.5 mg ciprofloxacin, twice daily	28 days	Inhalation	[98]
*P. aeruginosa* and/or *S. aureus*	Rhinosinusitis	3 drops ofloxacin 0.3%, three times daily	28 days	Nasal drops	[99]
*S. aureus*	Wounds	Mupirocin 2% ointment	-	Cutaneous	[100]
*S. aureus*	Catheters	50 mg/mL daptomycin	24 h	Catheter lumen	[101]
		10 mg/mL tigecycline			
		10 mg/mL rifampicin			
		10 mg/mL cotrimoxazole + 2500 U/mL heparin	12–24 h	Catheter lumen	[102]
		Minocycline-rifampin	-	Coating	[28]
*K. pneumoniae*	Catheters	doripenem and tobramycin	-	Catheter lumen	[103]
*P. aeruginosa*	Orthopedic procedures	1 g tobramycin + 12 or 24 MU colistin + 40 g polymethylmethacrylate	-	Intraoperative (PMMA beads)	[104]
*S. aureus*	Orthopedic procedures	40 mg/mL tobramycin + 1 g vancomycin + 10 mL packet of calcium sulfate	-	Intraoperative (calcium sulfate beads)	[104]
		2 mg/mL gentamicin aqueous solution	-	Intraoperative (injection)	[105]

**Table 5:** Examples of nanoparticles with anti-biofilm activities.

Type of Nanoparticles	Microbial Biofilm	References
Silver (immobilized on titanium)	*S. intermedius*	[112]
Silver	*P. aeruginosa*, *Shigella flexneri*, *S. aureus* and *S. pneumonia*	[115]
Titanium dioxide	*S. aureus* and *P. putida*	[116]
Selenium and selenium dioxide	*S. aureus*, *P. aeruginosa* and *Proteus mirabilis*	[117]
Zinc oxide and combination of zinc oxide and hydroxyapatite	*Streptococcus* sp.	[113]
Graphene oxide	*E. coli* and *S. aureus*	[107]

**Table 6:** Examples of diterpenoids with anti-biofilm activities.

Type of Diterpenoids	Microbial Biofilm	Mechanism of Action (Hypothetical)	References
Abietane (natural) salvipisone aethiopinone	*S. aureus*, *S. epidermidis*	NA	[14]
Abietane (synthetic) dehydroabietic acid scaffold with different amino acids	*S. aureus*, *S. epidermidis*	Bacterial membrane or the peptidoglycan (PG) layer	[81]
Diterpene serrulatane compound 8-hydroxyserrulat-14-en-19-oic acid (EN4)	*S. aureus* (methicillin-susceptible and methicillin-resistant), *S. epidermidis*	Membranolytic properties as well as a general inhibition of macromolecular biosynthesis	[83]

**Table 7:** Examples of biomacromolecules with anti-biofilm activities.

Type of Macromolecule	Microbial Biofilm	Mechanism of Action (Hypothetical)	References
Polyether ether ketone–octafluoropentyl methacrylate surface	-	Reduced protein adsorption	[91]
AHL lactonase (AiiA), a metallo-beta-lactamase produced by *Bacillus* spp.	*Vibrio cholerae*	blocks quorum sensing in Gram-negative bacteria by hydrolyzing N-acyl-homoserine lactones (AHLs)	[89]
Chitosan-based surface coating	*S. aureus*, *S. epidermidis*	anti-adhesive and bactericidal via contact membrane disruption	[92]
*K. pneumoniae* capsular polysaccharide	*S. aureus*, *S. epidermidis*, *E. coli*	NA	[87]
Commercially available organic acid water additives	*Salmonella Typhimurium biofilms*	Interference to intracellular pH homeostasis, membrane structure, osmolality and macromolecule synthesis	[90]
Synthetic PDMEA MeI polymers	*S. epidermidis*, *S. aureus*, *E. coli* and *P. aeruginosa*, *Candida albicans*	Membrane disruption	[84]

**Table 8:** Examples of honey with anti-biofilm activity.

Type of Honey	Microbial Biofilm	Source
Manuka	*P. aeruginosa*, *Streptococcus pyogenes*, *S. aureus*, *Klebsiella* spp., *Proteus mirabilis* *, *E. coli*, *Acinetobacter baumannii*, *Clostridium difficile*	[141,142,143,144,145,146,147,148]
Clover	*P. aeruginosa*, *S. aureus*, *Klebsiella* spp., *Proteus mirabilis*	[141,149]
Pumpkin	*Bacillus subtilis* (*B. subtilis*)	[150]
Chestnut and thyme	*B. subtilis*, *S. aureus*	[150]
Euphorbia	*B. subtilis*, *S. aureus*, *P. aeruginosa*, *E. coli*	[150,151]
Chaste	*B. subtilis*, *S. aureus*, *S. epidermis*	[150]
Multifloral	*B. subtilis*, *S. aureus*, *S. epidermis* *Staphylococcus mutans* *Listeria monocytogenes*	[150]
Eucalyptus	*B. subtilis*, *S. aureus*	[150]
Honeydew	*B. subtilis*, *S. aureus*, *S. epidermis*, *S. aureus*, *S. agalactiae*, *P. aeruginosa*, *E. faecalis* *	[145,150]
Lavender, strawberry and citrus	*P. aeruginosa*, *S. aureus* & MRSA	[152]
Sidr	*P. aeruginosa*, *S. aureus*, *E. coli*	[151]

* With a certain degree of resistance.
